# Clinical Application of Magnetic Resonance Imaging in Management of Breast Cancer Patients Receiving Neoadjuvant Chemotherapy

**DOI:** 10.1155/2013/348167

**Published:** 2013-06-05

**Authors:** Jeon-Hor Chen, Min-Ying Su

**Affiliations:** ^1^Center for Functional Onco-Imaging, Department of Radiological Sciences, University of California, Irvine, CA 92697-5020, USA; ^2^Department of Radiology, E-Da Hospital and I-Shou University, Kaohsiung 82445, Taiwan

## Abstract

Neoadjuvant chemotherapy (NAC), also termed primary, induction, or preoperative chemotherapy, is traditionally used to downstage inoperable breast cancer. In recent years it has been increasingly used for patients who have operable cancers in order to facilitate breast-conserving surgery, achieve better cosmetic outcome, and improve prognosis by reaching pathologic complete response (pCR). Many studies have demonstrated that magnetic resonance imaging (MRI) can assess residual tumor size after NAC, and that provides critical information for planning of the optimal surgery. NAC also allows for timely adjustment of administered drugs based on response, so ineffective regimens could be terminated early to spare patients from unnecessary toxicity while allowing other effective regimens to work sooner. This review article summarizes the clinical application of MRI during NAC. The use of different MR imaging methods, including dynamic contrast-enhanced MRI, proton MR spectroscopy, and diffusion-weighted MRI, to monitor and evaluate the NAC response, as well as how changes of parameters measured at an early time after initiation of a drug regimen can predict final treatment outcome, are reviewed. MRI has been proven a valuable tool and will continue to provide important information facilitating individualized image-guided treatment and personalized management for breast cancer patients undergoing NAC.

## 1. Clinical Significance and Concerns of NAC 

Neoadjuvant chemotherapy (NAC) has become an important alternative treatment modality for breast cancer. NAC can downstage cancers and render them operable and/or facilitate breast-conserving surgery (BCS) [1–4]. In patients with inoperable locally advanced breast cancer, NAC is the standard of care and has been shown to improve both disease-free survival and overall survival. Patients with operable cancer may also choose to receive NAC to facilitate BCS [4–6]. In a meta-analysis of 14 randomized trials consisting of 5,500 women comparing NAC first followed by surgery and surgery first followed by adjuvant chemotherapy for operable breast cancer, it was found that, although overall survival was equivalent in these two groups, the mastectomy rate was lower in the NAC group without hampering local control. NAC was also associated with fewer adverse effects [5].

**Figure 1:**
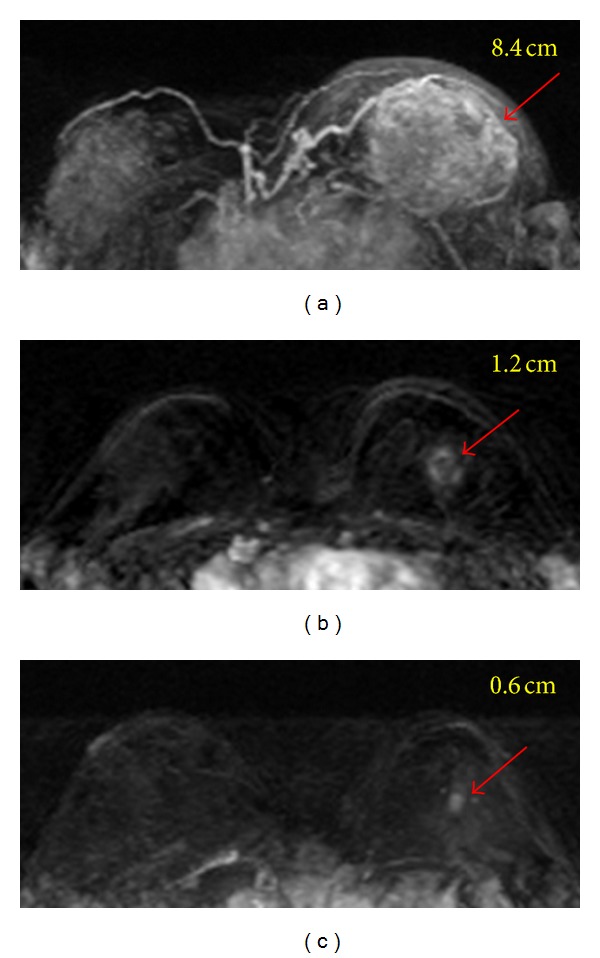
A 35-year-old patient with invasive ductal cancer in the left breast. From (a) to (c), the maximum intensity projection (MIP) images of pretreatment, F/U-1, and F/U-2 MRI are shown. Despite the large tumor, the boundary is clearly visible and this is a mass lesion. The diagnosed tumor size before treatment is 8.4 cm. This cancer is considered as inoperable and is recommended to receive NAC. After 2 cycles of treatment the tumor has shrunk to 1.2 cm, and the size is further decreased to 0.6 cm after completing NAC. Without NAC this patient will need mastectomy and will have a high risk of positive margin. NAC allows this patient to receive breast-conserving surgery, with a good cosmetic outcome.

As more effective therapies have become available, the main target of NAC has gone beyond down staging to a more far-reaching purpose of achieving pathological complete response (pCR). Emerging evidence suggests that induction of a pCR, or minimal residual cancer burden near pCR, is predictive of favorable long-term survival [6–8]. Depending on the treatment protocol, different chemo-regimens are used in combination or in a sequential order. A complete course of NAC usually takes several months. Patients receiving NAC do not always respond well. If a patient is not responding well to a certain regimen, the oncologist may change the drugs timely, not only to avoid unnecessary drug-related toxicity and complications, but also to allow the new regimen to work sooner. It is therefore critical to find a reliable method for assessing patient's response at an earlier time. With more effective therapy regimens, even large locally advanced tumors can be treated to achieve pCR or minimal residual disease, and as such another important role of imaging is to predict the residual disease after NAC so the results can be used for surgical planning. When MRI can confidently diagnose that the cancer has completely remised or shrunk to a minimum disease, a small lumpectomy is usually sufficient, and that will likely lead to a good cosmetic outcome after surgery. An example of how NAC can change the surgery from mastectomy to lumpectomy is given in Figure 1.

Research evidence has suggested that BCS after NAC results in acceptable low rates of locoregional or ipsilateral recurrence in appropriately selected patients, even in those with T3/T4 or multifocal/multicentric cancers [9–11]. Factors that predict early recurrence include residual pathologic tumor size >2 cm, multifocal tumor, and lymphovascular invasion [12]. Therefore, an accurate pre- and post-NAC disease staging is very important for selecting the optimal patients suitable for breast-conserving surgeries, without subjecting them to the high risk of recurrence [9–15]. Current methods for assessing treatment response include clinical examination (palpation), sonography, mammography, MRI, and molecular imaging. Because the reliability of traditional methods (physical examination, mammography, and ultrasonography) is questionable [13–16], MRI is increasingly being used to evaluate response of breast cancer undergoing NAC. MRI-measured tumor size after NAC has been proven to be well correlated with pathologically determined tumor size after completing therapy, and early change of tumor size has been shown to be a good response indicator [16–19]. However, changes in lesion size on MRI are usually not detected until several weeks following chemotherapy [18]. If early surrogate response indicator could be established to predict final treatment outcome, it would help to allow timely adjustment of drug regimens and achieve the goal of pCR.

## 2. Breast MRI Methods for NAC Response Evaluation

Dynamic contrast-enhanced MRI (DCE-MRI) is the current standard for breast MR imaging. The enhancement kinetics can be evaluated using 3 distinct features, the wash-in phase, the maximum enhancement, and the wash-out phase. Several heuristic parameters can be analyzed from the curve, such as wash-in slope (maximum slope, or the slope within a time period), the maximum percent enhancement, time to maximum, and the wash-out slope (within a time period). A more sophisticated analysis method is to perform pharmacokinetic analysis based on two compartmental models, such as the widely used unified Tofts model [20, 21]. The two compartments are the vascular space and the interstitial space (or the extravascular-extracellular space), with the transfer constant *K* ^trans⁡^ to leak from the vascular to the interstitial space and the rate constant *k*_ep_ from the interstitial space back to the vascular space. In addition to diagnosis, another major application of DCE-MRI is for predicting response of breast cancer undergoing NAC. It is well known that the cancer therapy also causes vascular damage, and the enhancement kinetic pattern will change from the wash-out pattern to a less aggressive pattern of plateau or persistent enhancement [22]. In general, when there is tissue enhancement within the previous tumor bed after NAC, it is considered as residual disease regardless of the DCE kinetic pattern. 

There are attempts to investigate whether information provided by MRI may serve as earlier response indicators than the size change. The parameters included percent enhancements measured at different times during the DCE imaging period, initial area under the curve, and pharmacokinetic parameters (such as *K* ^trans⁡^ and *k*_ep_) measured by DCE-MRI; apparent diffusion coefficient (ADC) measured by diffusion-weighted imaging (DWI); and choline and water : fat ratio measured by proton MR spectroscopy (MRS). These imaging parameters are measured in pretreatment MRI and early follow-up MRI (after one or two cycles of NAC) to predict the final response. Final MRI following the completeness of NAC treatment is very important in evaluating the residual tumor size hence an optimal surgical plan can be chosen. Besides focusing on evaluation of tumor itself, recently, there has been increasing interest in the evaluation of normal breast tissue, such as background parenchymal enhancement [23–30] and breast density [31]. In the following sections we will describe the measurement of these imaging parameters at different times during NAC and their clinical roles in improving management of patients.

## 3. Pretreatment MRI for Predicting NAC Response and Prognosis

Most NAC imaging studies focused on evaluating posttreatment responses. However, the features of tumors in pretreatment MRI are known to be associated with treatment responses. It was noted that large tumor size, a diffuse lesion without mass effect, and high intratumoral signal intensity on T2-weighted MR images were significantly associated with chemoresistance. Mass lesions showing the wash-out DCE kinetic pattern were significantly associated with chemosensitivity [32]. Similarly, in a study of triple negative tumors, it was noted that an irregularly shaped lesion (*P* = 0.018) and the presence of clear intra-tumoral necrosis (*P* = 0.044) were significantly associated with poor NAC response [33]. In a meta-analysis of nine studies, it was noted that several pretreatment MR parameters could differentiate between responders and nonresponders [34]. Predictive role of these parameters measured using different MR imaging methods will be described later in subsections.

Pretreatment DCE-MRI parameters had also been used to predict disease-free and overall survival for breast cancer patients receiving NAC [35]. Overall, a more aggressive disease with a larger tumor and higher angiogenic properties was associated with worse prognosis. It was noted that, in patients who exhibit high levels of vascular perfusion and permeability in pretreatment DCE-MRI, significantly lower disease-free survival (DFS) and overall survival (OS) are expected [35]. Univariate survival analysis has revealed that certain empirical DCE-MRI parameters (including maximum enhancement, enhancement at an early time, wash-in slope, and area under the initial curve) showed significant association with both DFS and OS [35]. In another study, a significant correlation between the total enhancing tumor volume and 5-year survival was found (*P* < 0.05) [36]. It was shown that a two-dimensional discriminator considering both the total enhancing tumor volume and the tumor volume showing wash-out DCE pattern further improved the prediction of survival, with *P* < 0.001 differentiating between survivors and nonsurvivors [36]. Similarly, in a study of 62 patients, pretreatment extravascular extracellular volume *V*_*e*_ (*P* = 0.027) and mean transit time (MTT) (*P* = 0.002) were associated with disease-free survival [37]. 

## 4. Early Response Predictors Using Different MR Imaging Methods

### 4.1. DCE-MRI

**Figure 2:**
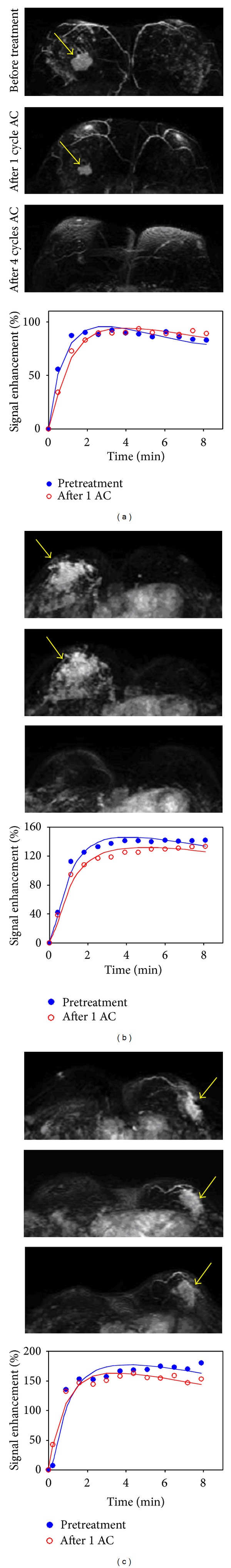
The DCE kinetics measured at baseline before treatment and after 1 cycle of chemotherapy from 3 case examples. From top to bottom, the maximum intensity projection (MIP) images of pretreatment, F/U-1 MRI after 1 cycle of treatment, and F/U-2 MRI after 4 cycles of treatment are shown. (a) A responder after 1 cycle of AC regimen, which shows a slower wash-in and a slower wash-out after chemotherapy. (b) A confirmed responder after 4 cycles which has not yet shown a good response after 1 cycle, but the change of DCE kinetic is similar to the responder in (a) thus indicating that it may be a responder. The follow-up MRI is performed 8 days after the administration of chemotherapy, which may be too early to show size change. (c) A nonresponder which does not show tumor shrinkage after 4 cycles of AC treatment, and the DCE shows a faster wash-in and a faster wash-out after 1 cycle of chemotherapy. However, despite the noticeable differences in the DCE patterns, the changes are subtle.

There is discrepancy in the published data regarding the usefulness of pharmacokinetic parameters in predicting NAC response [38–41]. Some found that they could predict final response earlier than the size measurement did [40], but others did not [39, 41]. The explanation for these variations in reported data is multifactorial: patient number, tumor type, chemotherapeutic agent, the follow-up imaging time of MRI after commencing therapy, and the analysis methods, have all varied. Yu et al. [41] found that the changes of *K* ^trans⁡^ or *k*_ep_ after one cycle of AC by itself could not provide better information than the early tumor size change to predict response, but they could be combined with size change to better differentiate responders from nonresponders.  Figure 2 shows 3 case examples. In this study by Yu et al., the DCE kinetics was measured by manually drawing a region of interest (ROI) based on the enhanced tumor, and only subtle changes in the DCE patterns were noted between pretreatment and after 1 cycle of AC follow-up MRI, but these changes could not differentiate between responders and non-responders after completing 4 cycles of AC. It was concluded that the followup performed soon after 1 cycle of AC (within 2 weeks after starting of treatment) was too early. Another study showed significant early reduction in both *K* ^trans⁡^ and *k*_ep_ in responders compared to non-responders [40]. Changes in *V*_*e*_ and *K* ^trans⁡^ were significantly different between non-, partial-, and complete responders (*P* = 0.009 and *P* = 0.04, resp.) [42]. Breast tumor is highly heterogeneous and the ROI-based analysis cannot provide detailed information about the responses in different parts of tumor. Therefore, for the purpose of evaluating therapeutic changes, the most useful analysis method is to perform pixel-by-pixel analysis of the DCE enhancement kinetics, and the obtained histograms for the analyzed parameters can be compared between studies performed before and after therapy to evaluate changes [22, 39].

Other than predicting the efficacy of cytotoxic chemotherapy, the more attractive role of DCE-MRI is to evaluate the response of antiangiogenic or antivascular therapy [43–48]. Evidence from phase I and II studies strongly suggests that *K* ^trans⁡^ can be used as a predictive biomarker to determine response to antiangiogenic drugs or vascular disruptive agents, with a change in *K* ^trans⁡^ of greater than 40% considered as the threshold required to represent definitive response [49]. The most widely used antiangiogenic agent, trastuzumab (Avastin), is a monoclonal antibody that neutralizes the vascular endothelial growth factor (VEGF) to inhibit angiogenesis, and it has been used for treating breast cancer in neoadjuvant setting. DCE-MRI provides a means for assessing the treatment-induced vascular changes to investigate the early therapeutic response to this targeted drug [50]. It may provide insightful information to evaluate the efficacy of drugs in clinical trial phases and to guide the design for future studies. However, since bevacizumab was often combined with chemotherapy for breast cancer treatment, the combined cytotoxic and antiangiogenic effects were observed, and as such the role of DCE-MRI for predicting the sole efficacy of bevacizumab could not be established. 

### 4.2. Proton MR Spectroscopy

**Figure 3:**
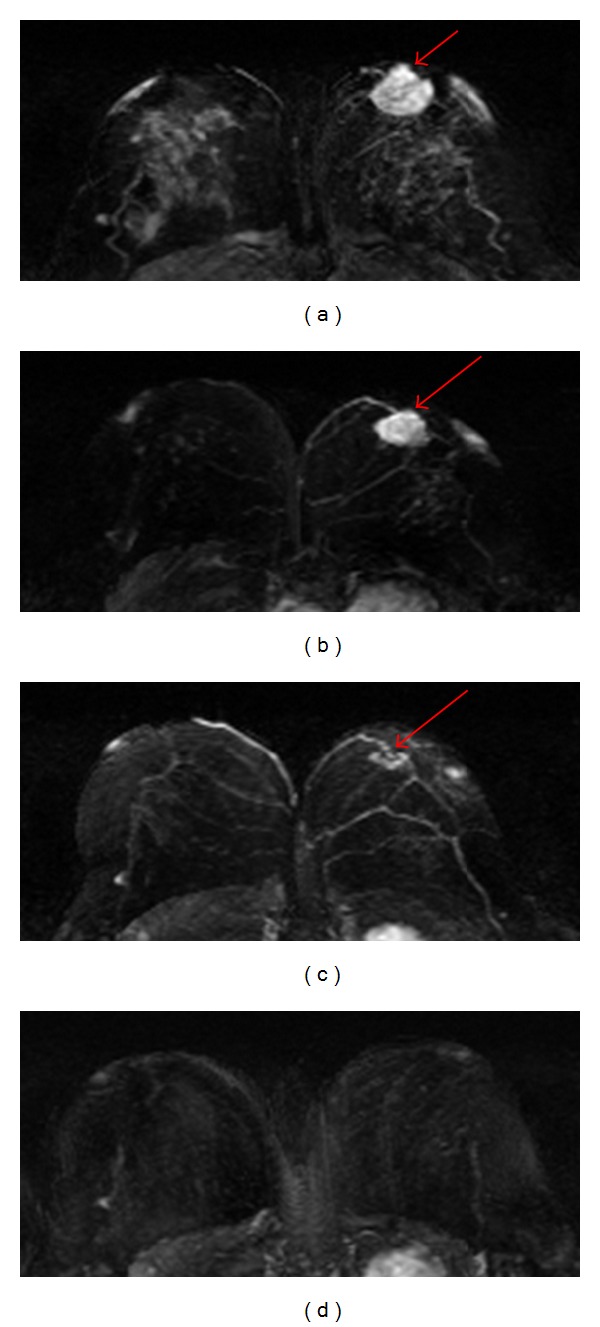
A 41-year-old patient with a mass lesion (invasive ductal cancer) in the left breast. From (a) to (d), the maximum intensity projection (MIP) images of pretreatment, F/U-1, F/U-2, and F/U-3 MRI are shown. The tumor size is 4.0 cm before treatment, which shrinks down to 2.7 cm in F/U-1 (32% reduction), 1.2 cm in F/U-2, and reaches a complete response in F/U-3 after completing NAC. This patient is confirmed as pCR in post-NAC pathological examination. The total choline concentration measured by MRS is [tCho] = 2.33 ± 0.54 mmol/kg before therapy, which decreases to 1.15 ± 0.25 mmol/kg in F/U-1, showing 51% reduction. Tumor size is too small in F/U-2 and F/U-3 for MRS measurements. For this pCR case, the tumor size reduction is 32% at F/U-1, and [tCho] reduction is greater at 51%.

**Figure 4:**
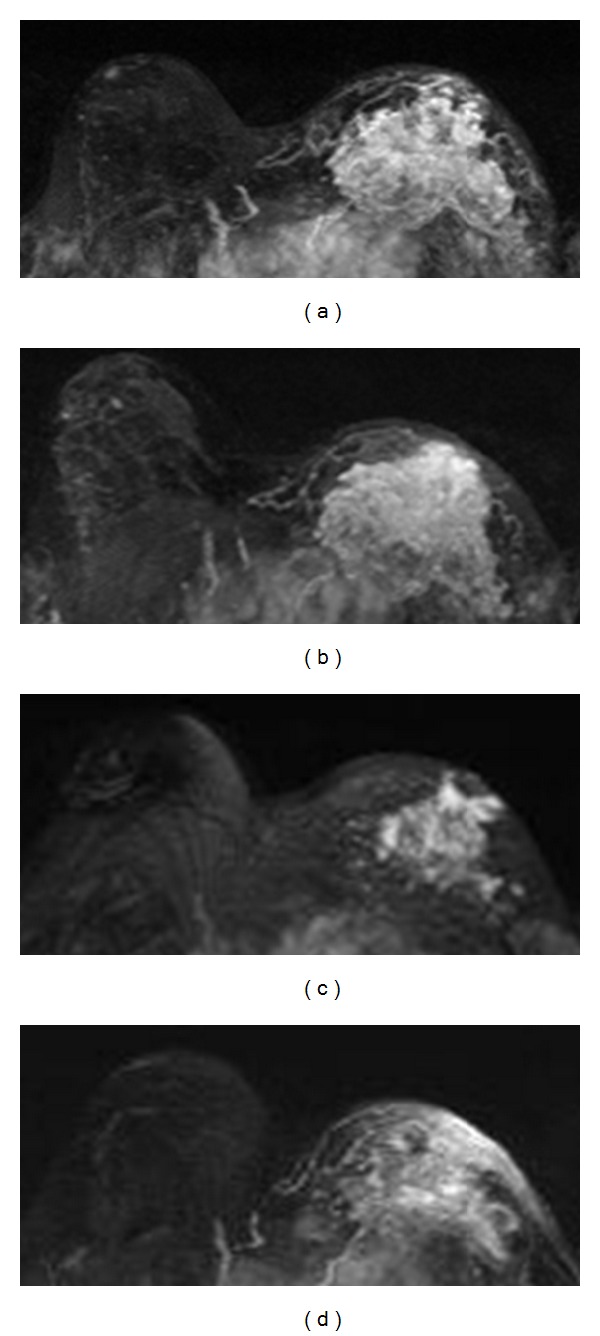
A 29-year-old patient with non-mass-like enhancement lesion in the left breast. From (a) to (d), the maximum intensity projection (MIP) images of pretreatment, F/U-1, F/U-2, and F/U-3 MRI are shown. The extent of tumor size is 8.2 cm before treatment, remains about the same at 8.0 cm in F/U-1, shrinks down to 4.5 cm in F/U-2, and progresses again to 6.2 cm in F/U-3. The choline measured by MRS shows [tCho] = 0.77 ± 0.11 mmol/kg before treatment, which decreases to 0.20 mmol/kg in F/U-1, and then increases to 1.01 mmol/kg in F/U-2, and further increases to 1.70 mmol/kg in F/U-3. The transient decrease of tCho in F/U-1 precedes the size reduction observed later in F/U-2. And then the increase of tCho in F/U-2 indicates treatment failure, and the tumor grows larger in F/U-3.

1H-MRS can be used to detect the elevated choline concentration in breast cancer. High levels of choline-containing metabolites (referred to as total choline or tCho) are mainly due to the increase of phospholipid metabolism and cellular membranes proliferation. Many studies have investigated the role of 1H-MRS for therapy response prediction but inconsistent results were reported [51–58]. Therefore, the value of MRS was not well established, partly due to its technical difficulty in quantification [51–53]. In early studies, Kvistad et al. [51] and Jagannathan et al. [52] demonstrated that 1H-MRS at 1.5 T was useful to assess the response of locally advanced breast cancer to NAC. They, however, used qualitative observations not quantitative measurements of tCho concentration to monitor changes. Meisamy et al. [53] reported a quantitative 1H-MRS study in 13 patients using a 4.0 T scanner and found that the change in tCho level within 24 hours was significantly different between the responder and the nonresponder groups; however, this result could not be further verified by other studies. Baek et al. reported significant difference in tCho level between clinical responders and non-responders evaluated based on the size changes at a later time [54]. In a follow-up study by Baek et al. [55] using pathologic response as the outcome, it was found that the tCho changes were greater than the tumor size changes in the pCR group in both F/U-1 (after 3-4 weeks) and F/U-2 (after 6–8 weeks) studies but not in non-PCR group. The results suggested that, when the tCho reduction was higher than the tumor size reduction, the tumor was more likely to achieve pCR [55]. However, as the treatment continues, the change in tumor size halfway through therapy (6–8 weeks) was the most accurate predictor of pCR, with area under the ROC curve of 0.9, while that for the change in tCho was 0.73. Example of a pCR case is shown in Figure 3, and a non-pCR case is shown in Figure 4. Tozaki et al. showed that, after one cycle of chemotherapy, a reduction in the choline signal was more sensitive than DW-MRI in demonstrating pathological response [56, 57]. It was also shown that the changes in Cho after the second cycle of chemotherapy may be more sensitive than changes in the tumor size to predict the pathological response [56]. Another study found that a significant decrease in tCho SNR was detected after treatment, but responders could not be distinguished from non-responders [58]. It was concluded that, with the currently observed low choline detection rate, technological challenges related to choline detection have to be resolved before MRS can provide a reliable quantitative imaging biomarker for predicting NAC response [58].

Performing MRS quantification over a course of treatment is particularly challenging because it is known that water content and T2 vary under normal physiological conditions [38]. In addition, as the lesion shrinks, it is more difficult to quantify tCho because there is less tumor tissue to be measured. This is an inherent problem with the relatively low sensitivity of 1H-MRS compared with MRI, which limits the utility of 1H-MRS. Further work is also necessary to account for the changes of water T2 relaxation rate, which also decreases in successful therapy. Other than tCho, several studies also reported an association between the water : fat ratio measured by MR spectroscopy with NAC response [38, 59–61]. As tumor shrinks, the water content will decrease, and the ratio to the fat content may serve as a response indicator. Manton et al. found that, while pharmacokinetic parameters and ADC could not detect early treatment response, early changes in water : fat ratios and water T2 relaxation time did demonstrate substantial prognostic efficacy after two cycles of NAC [38]. However, many factors other than tumor response may also affect the water : fat ratios and water T2 measurements, and these two parameters were not considered as reliable response indicators either.

### 4.3. Diffusion-Weighted Imaging

DW-MRI is developed to probe the microscopic motion of water molecules, and the measured apparent diffusion coefficient (ADC) is sensitive to cell density, membrane integrity, and tissue microstructure [62]. Tumors, in general, have a high cell density with restricted water diffusion. The decease of cellular density after NAC will lead to increased ADC, and that shows promise as an early surrogate biomarker for detecting early response before tumor shrinkage occurs. In a meta-analysis of 6 studies, DW-MRI sensitivity was 0.93 (95% CI 0.82–0.97) and specificity was 0.82 (95% CI 0.70–0.90) [63] in predicting pathological response. Induction of successful apoptosis will result in loss of cell membrane integrity, and the altered barrier will allow more free water diffusion, which can be used as a very early sign of treatment response. The later cell death and shrinkage will increase extracellular space, which translates to a rise in the ADC value of up to 35% [64–66]. 

The initial results using ADC as a predictor were encouraging, showing earlier change than size reduction [64–67]. It was noted that the change in ADC after the first cycle was statistically significant compared with the change in tumor volume or diameter [65]. The coupling of the diffusion imaging with the established morphological MRI provides superior evaluation of response to NAC compared with morphological MRI alone [68]. Changes in MRI-derived tumor diameter and ADC after only one cycle of NAC could provide a valuable tool for early evaluation of treatment effects [69, 70]. ADC measured after four cycles of NAC was shown to be a strong independent predictor of pCR [71]. After 3–6 cycles of NAC, the best cut-off for differentiating pCR from non-pCR was a 54.9% increase in the ADC, which could reach 100% sensitivity and 70.4% (19/27) specificity [72]. However, while ADC can be precisely measured for mass type lesions, it is difficult for lesions that present as non-mass-like enhancements, and it is challenging to use ADC to predict the NAC response for non-mass type lesions [70]. 

Breast cancer with a low pretreatment ADC tended to respond better to chemotherapy [73, 74]. There was a significant negative correlation between pre-chemotherapy ADC and the percentage change of tumor volume [74]. It was noted that high ADC values indicate necrotic tissue with low cellularity [75, 76]. Necrotic areas in tumors are usually poorly perfused, which may reduce the delivery of chemotherapeutic agents to the tumor. Furthermore, tumor tissues near necrotic regions are likely in hypoxic status and have slower metabolisms and thus less sensitive to cytotoxic chemotherapy [73]. However, other studies did not find significant difference in pre-chemotherapy ADCs between pathologic complete response cases and those with residual diseases [66, 77]. The conflicting findings may be owing to different DWI acquisition methods (e.g., *b* value, fat suppression technique) and methodological differences in measurements of ADC and residual tumor size used in the different studies [74]. The optimal *b* values for diffusion-weighted MRI in the breast have not been established yet. While there was a standard recommended protocol for DCE-MRI of the breast, there has been no guideline for the DWI scanning protocol. The imaging parameters and analysis methods all have a bearing on the measurement of ADC values [66]. Further studies are needed to standardize the protocol, so that the measured ADC values can be compared across different studies. 

## 5. Accuracy of MRI in Determining Residual Disease after Completing NAC

**Figure 5:**
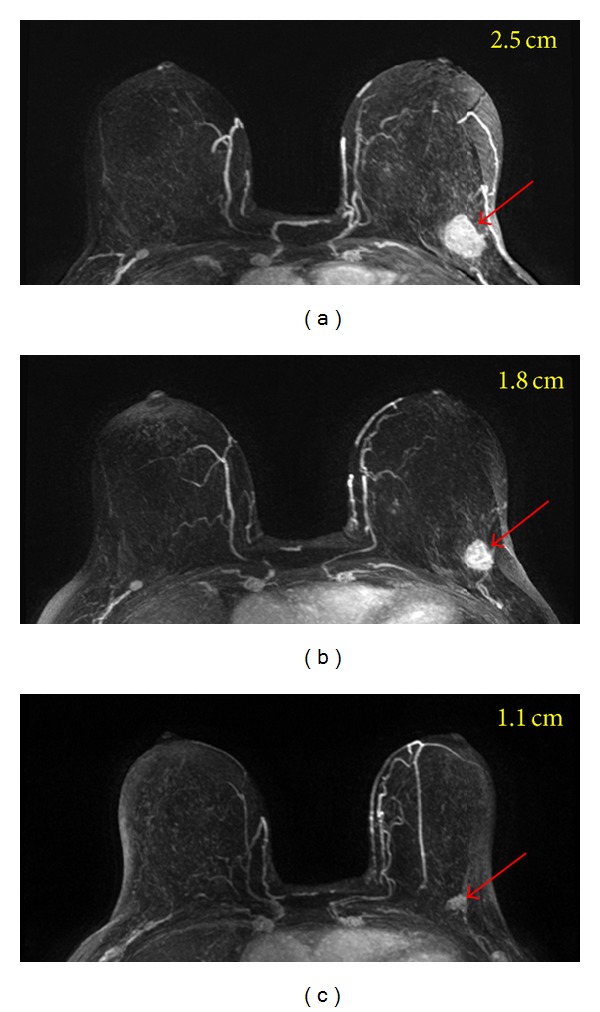
A 64-year-old patient with a well-circumscribed mass lesion (invasive ductal cancer) in the left breast. From (a) to (c), the maximum intensity projection (MIP) images of pretreatment, F/U-1, and F/U-2 MRI are shown. The tumor size is 2.5 cm before treatment and shows concentric shrinkage to 1.8 cm in F/U-1 and further down to 1.1 cm in F/U-2 after completing treatment. The residual tumor size determined in post-NAC pathological examination is 1.4 cm. For mass lesion that shows concentric shrinkage, MRI is accurate in diagnosing residual disease.

**Figure 6:**
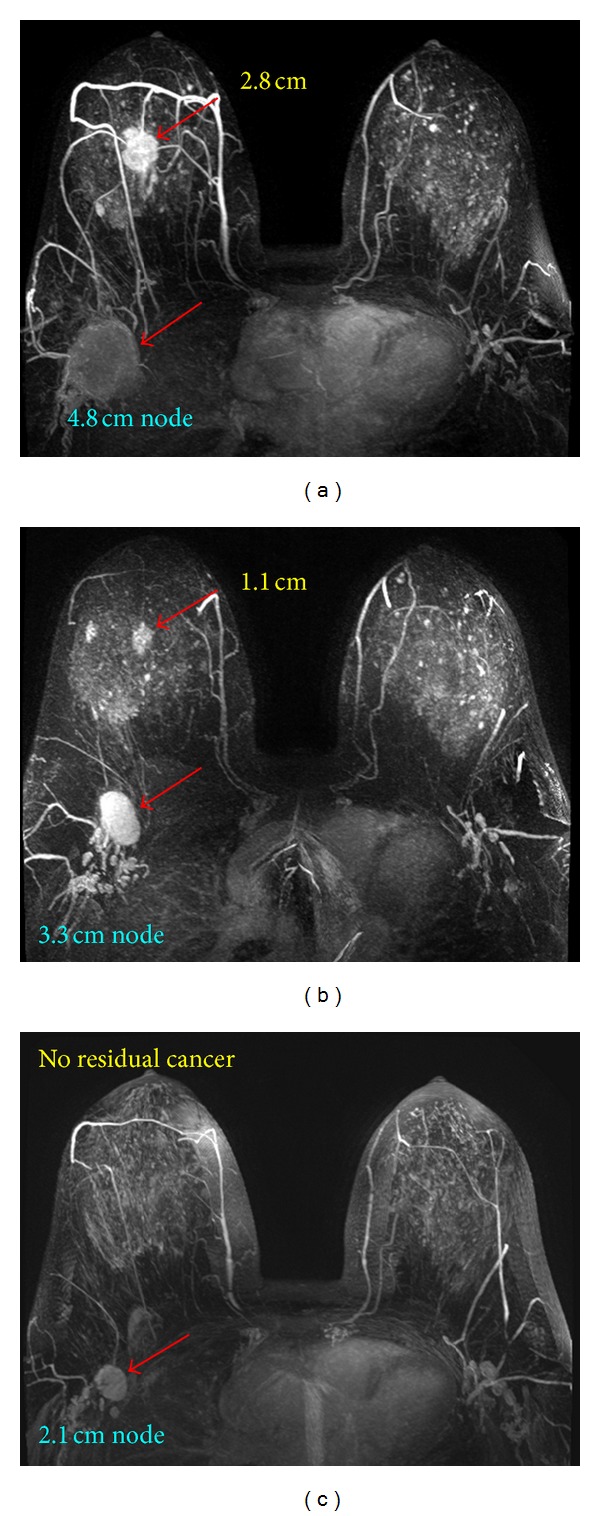
A 48-year-old patient with a mass lesion (invasive ductal cancer) in the right breast and an enlarged lymph node in the axilla. From (a) to (c), the maximum intensity projection (MIP) images of pretreatment, F/U-1, and F/U-2 MRI are shown. The size of the primary tumor in the breast is 2.8 cm before treatment, which shrinks down to 1.1 cm in F/U-1 and reaches a complete response in F/U-2 after completing NAC. The node is also responding well and shows size shrinkage from 4.8 cm before treatment to 3.3 cm in F/U-1 and to 2.1 cm in F/U-2. In addition to evaluating the response of primary tumor, MRI can also be used to evaluate the response in the nodes. This patient is confirmed to reach pCR in the post-NAC pathological examination, with one positive node.

**Figure 7:**
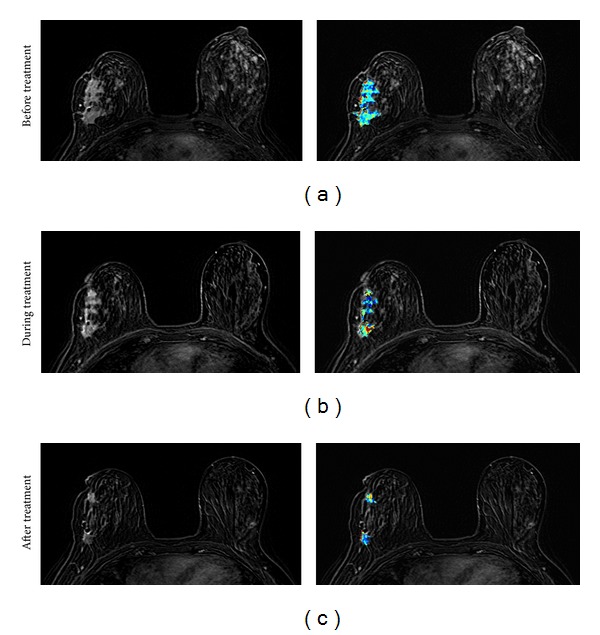
A 41-year-old patient with non-mass-like enhancement lesion (invasive ductal cancer with lobular features). From (a) to (c), the contrast-enhanced (subtraction) images selected from the same level in pretreatment, F/U-1, and F/U-2 MRI are shown. The tumor boundary cannot be clearly determined, and thus the extent of the tumor cannot be measured precisely. While the disease extent does not change much in F/U-1, it is noticeable that the area of the enhanced tissues is smaller in F/U-2 after completing NAC. The right panel shows the corresponding color-coded *K* ^trans⁡^ maps analyzed using pixel-by-pixel pharmacokinetic analysis within the enhanced tumor area, based on the unified Tofts model. It can be seen that the tumor breaks into two areas that show strong enhancements in F/U-2. The post-NAC pathological examination shows nearly continuous cancer clusters within a 6.5 cm region. It is typical for a non-mass lesion to show scattered diseases within the original tumor bed.

**Figure 8:**
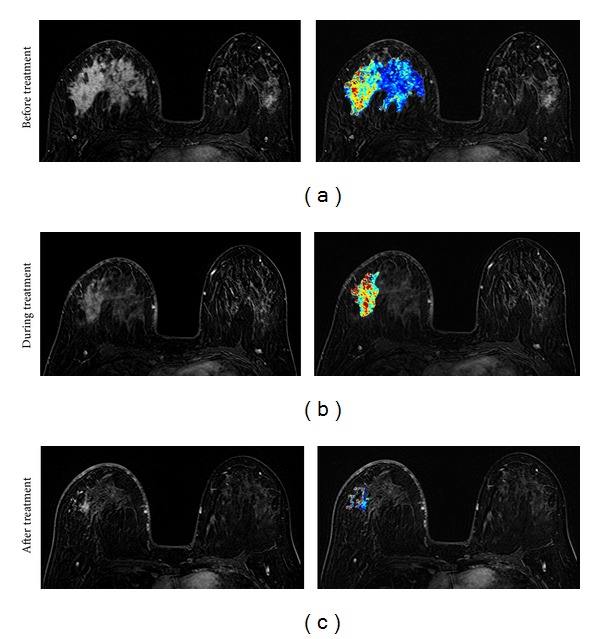
A 31-year-old patient with non-mass-like enhancement lesion (invasive ductal cancer with extensive carcinoma in situ components). From (a) to (c), the contrast-enhanced (subtraction) images selected from the same level in pretreatment, F/U-1, and F/U-2 MRI are shown. The tumor boundary cannot be clearly determined, and thus the extent of the tumor cannot be measured precisely. The area of the enhanced tumor tissues and the degree of enhancement are decreasing with treatment, indicating a good response to the chemotherapy. The right panel shows the corresponding color-coded *K* ^trans⁡^ maps analyzed using pixel-by-pixel pharmacokinetic analysis. The post-NAC pathological examination shows scattered cancer cells within a 10 cm region. It is typical for a non-mass lesion to show scattered diseases within the original tumor bed. Despite the decreased cancer cell density responding to NAC, this patient still needs mastectomy.

Many studies have investigated the role of breast MRI as a diagnostic tool for evaluating the extent of residual disease after NAC [78, 79]. Despite the superior accuracy when compared with other modalities, MRI can over- or underestimate residual tumor extent. This inaccurate assessment may be influenced by tumor response, chemotherapeutic agent, or NAC-induced reactive changes within the tumor [80]. The general agreement is that MRI is very accurate for mass type lesions that show clear tumor boundary and present concentric shrinkage after therapy (Figures 5 and 6). In contrast, MRI is not accurate for non-mass-like enhancement lesions that are more likely to break up into pieces and present residual disease as scattered cells or cell clusters (Figures 7 and 8). Invasive lobular cancers and cancers with extensive ductal carcinoma in situ components are more likely to present non-mass type lesions, and the accuracy of MRI may be compromised [81, 82]. 

Another observation is that the accuracy of MRI is affected by the molecular characteristics of cancer [81–90]. HER-2-positive cancer is more aggressive and there is targeted therapy trastuzumab (Herceptin) available; therefore, HER-2-positive cancer generally responds very well to trastuzumab-containing chemotherapy [81]. When the treatment is more effective, the rate of achieving pCR is higher, and it is less likely to present the scattered minimal residual disease confounding the accuracy of MRI diagnosis. Therefore, the diagnostic accuracy of post-NAC MRI is generally better in HER-2-positive-than in HER-2-negative cancer. MRI is known to have a high false-negative rate in HER-2-negative patients [81]. For HER-2-negative patients receiving NAC with and without bevacizumab, the pathological response and the diagnostic performance of MRI are comparable. In both groups, MRI has a limitation in detecting residual disease broken down to small foci and scattered cells/clusters [82].

Hormonal receptor status (including estrogen receptor ER and progesterone receptor PR) also affects the response to chemotherapy and thus the diagnostic accuracy of post-NAC MRI. In general, hormonal-negative cancer is more aggressive and responds better to chemotherapy, and as such the diagnostic accuracy of MRI in hormonal-negative cancer is better than in hormonal-positive cancer. It has been shown that MRI is more accurate in triple-negative or ER-negative/HER2-positive disease, but is less accurate in ER-positive/HER2-negative breast cancer [83–87]. For HER-2-negative- and hormonal-receptor-positive cancers, they are more likely to show residual disease as small foci or scattered cells after NAC leading to underestimation of residual disease extent on MRI [84]. Another study, however, showed that, after multivariate analysis, molecular subtype and systemic regimen administered did not significantly influence the sensitivity, specificity, PPV, or NPV of MRI in predicting pathologic response [88]. The morphological appearance of tumor (mass versus non-mass) may have a more profound influence on the MRI accuracy than the molecular subtypes. 

Since MRI done at 1.5 T showed a high false-negative diagnosis when the residual tumor was presenting as a scattered pattern with multiple small foci of invasive cancer cells distributed in a large area [81, 82], it raises a question about whether a higher spatial resolution using 3 T may improve the accuracy. A recent study has found that breast MR done at 3.0 T still has the same limitation as 1.5 T in detection of small and scattered tumor cell clusters after NAC [84]. The higher field at 3 T comes with worse field homogeneity and longer T1 relaxation time [91, 92] which may cause lower signal and show less contrast enhancements leading to false-negative diagnosis [93–95]. Nevertheless, 3 T with a higher spatial resolution and signal-to-noise ratio may reveal more significant findings compared to 1.5 T and provide an improved assessment of the response to NAC [96]. Recently, dedicated 7 T breast MRI is proven technically feasible for monitoring NAC [97]. As more ultrahigh field MRI scanners become available, it will be interesting to see how this may be used to improve the accuracy, particularly for the non-mass lesions that present scattered minimal disease after NAC.

Preliminary results using DWI in assessing residual tumor extent after completing NAC have been reported [77], which found that the accuracy for depicting residual tumor was 96% for DWI, compared with an accuracy of 89% for contrast-enhanced MR imaging (*P* = 0.06). The use of DW imaging to visualize residual breast cancer without the need for contrast medium could be advantageous in women with impaired renal function [77]. However, since the spatial resolution of DWI is often worse than that of DCE-MRI, the advancement of scanner technology, including better gradient coil with a higher strength and less geometrical distortion, is important for DWI to reliably diagnose residual disease after completing NAC. 

In patients who had more extensive pretreatment disease, despite an excellent response to NAC, the surgeons still tended to apply an aggressive approach and recommended mastectomy. Given that the confirmation of pCR or minimal residual disease would change surgeons' recommendations for less aggressive, conservation surgery, the maturity of MRI for NAC response prediction may provide reliable staging information to aid in the recommendation of the optimal surgical procedure [98].

## 6. Breast Stromal (Parenchymal) Enhancement Related to NAC

Background parenchymal enhancement refers to the enhancement of the normal breast glandular tissue. Age, menstrual or menopausal status, and hormonal use can affect breast glandular tissue enhancements [23–25, 30, 99, 100], and this normal tissue enhancement may impact the diagnostic performance of breast MRI [28, 99, 101–103]. The value of normal tissue enhancement in the diseased breast on MRI was noted to be associated with response to NAC [104]. Higher signal enhancement ratios in breast stroma after one cycle of chemotherapy are significantly associated with decreased local recurrence and longer disease-free survival [104]. A high stromal signal enhancement ratio may reflect greater microvessel density and thus better delivery of the chemotherapeutic agent to the tumor, which would result in a better clinical response and decreased likelihood of recurrence after surgery [104]. This research area is very new with little data, and more research is needed to investigate the significance of the background stromal tissue enhancement during the NAC treatment and prognosis. 

## 7. Reduction of Breast Density following NAC 

Breast density is a strong independent risk factor associated with the risk of developing breast cancer. It was found that an increase in BI-RADS density category within 3 years is associated with an increase in breast cancer risk and a decrease in density is associated with a decreased risk [105]. Change in mammographic breast density is an excellent predictor of response to tamoxifen in the preventive setting [106]. It was proven that women receiving tamoxifen and experiencing a 10% or greater reduction in breast density had 63% reduction in breast cancer risk (odds ratio = 0.37, 95% CI = 0.20 to 0.69, *P* = 0.002), whereas those who took tamoxifen but experienced less than a 10% reduction in breast density had no risk reduction (odds ratio = 1.13, 95% CI = 0.72 to 1.77, *P* = 0.60). 

**Figure 9:**
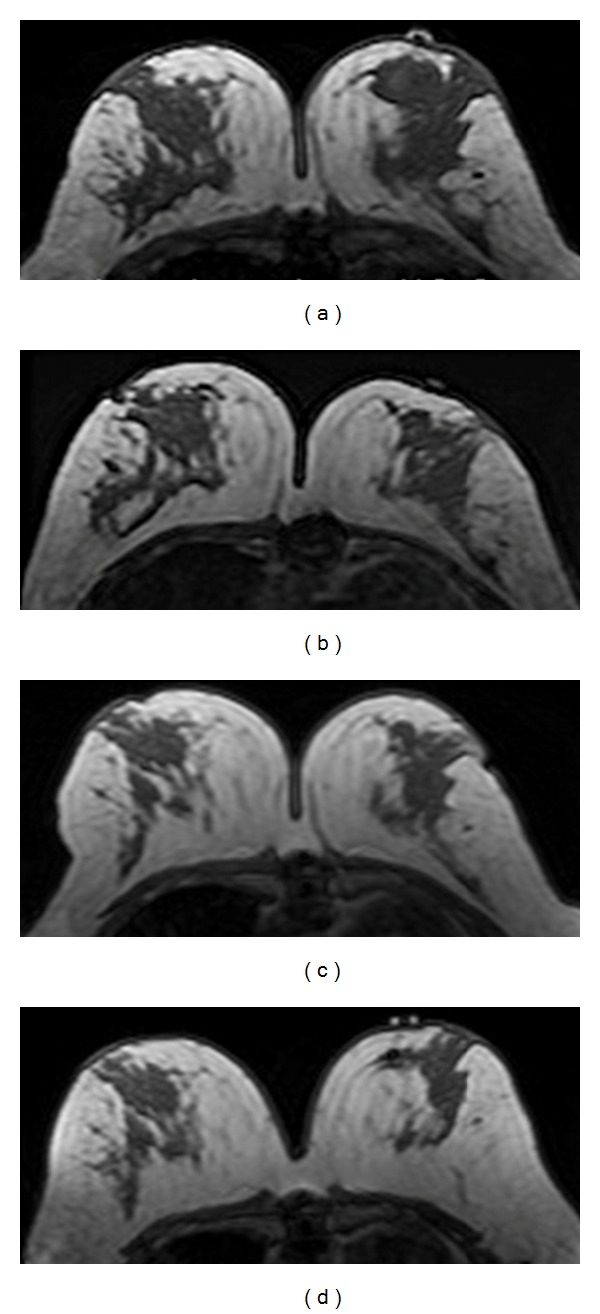
This is the same 41-year-old patient shown in Figure 3. The cancer is in the left breast, and the breast density is measured from the normal breast in the right side. From (a) to (d), the non-fat-sat T1-weighted images selected from the same level in pretreatment, F/U-1, F/U-2, and F/U-3 MRI are shown. It is noticed that the normal breast density decreases with chemotherapy. The measured percent density (fibroglandular tissue volume divided by the breast volume) is 13.4% before treatment, which decreases to 9.2% in F/U-1 after receiving 2 cycles of AC regimen and further down to 8.7% in F/U-2 and 8.2% in F/U-3.

After NAC, normal breast tissue shows significant atrophy of the terminal ductal lobular units [107]. This includes reduction of the lobular acini, lobular sclerosis, and the attenuation of the lobular/ductal epithelium. By using MR imaging, it was found that patients receiving NAC showed decreased breast density in the normal breast, and the effects were significant after initial treatment with one to two cycles of the AC regimen (Figure 9) [31]. Our recent findings (unpublished data) also find that the taxane-based regimen causes density atrophy in the normal breast. Since the density reduction was age-dependent (more pronounced in younger patient), the NAC-related density reduction was more likely mediated through the suppression of ovarian function [108, 109]. Whether this density change in the normal breast is associated with patient's prognosis and the future risk of developing contralateral breast cancer warrants further investigation.

## 8. Conclusion

In this review paper we summarized the clinical application of MRI in management of breast cancer patients undergoing NAC. As many patients may become good candidates for breast-conserving surgery, the most well-established role of MRI is to evaluate the extent of residual disease after NAC for surgical planning. During the NAC treatment, MRI can be performed at different times to evaluate the response to different drug regimens, and that provides opportunities for timely adjustment of treatment protocols to improve the chance of achieving pCR while avoiding unnecessary toxicity. DCE-MRI with a high spatial resolution and a good tissue contrast is essential to evaluate the change of tumor size, which is still the most reliable response indicator. Proton MR spectroscopy can detect early response based on the changes in choline or water : fat ratio, but the difficulty in quantification makes MRS not a reliable tool for predicting NAC response. Diffusion-weighted MRI has a great potential to provide early response indicator based on the altered cell membrane and cell death, but the relatively low spatial resolution and image distortion limit DWI to become a good tool for evaluating the extent of residual disease after NAC. The background parenchymal enhancement and the density of the normal breast tissue are two emerging parameters that are currently being investigated. These MR imaging parameters may also have a prognostic value to predict patient's disease-free and overall survival, which needs to be further established. Overall, MRI is a valuable imaging modality for evaluating the pretreatment disease, response during NAC, and the residual disease after completing NAC. With the continuing technology advancement and more widespread use of MRI, it will benefit many more breast cancer patients in the future by providing them with individualized image-guided treatment and personalized management.
